# Relative adrenal insufficiency in mice deficient in 5α-reductase 1

**DOI:** 10.1530/JOE-13-0563

**Published:** 2014-08

**Authors:** Dawn E W Livingstone, Emma M Di Rollo, Chenjing Yang, Lucy E Codrington, John A Mathews, Madina Kara, Katherine A Hughes, Christopher J Kenyon, Brian R Walker, Ruth Andrew

**Affiliations:** Endocrinology, Queen's Medical Research Institute, University and British Heart Foundation Centre for Cardiovascular Science, University of Edinburgh, 47 Little France Crescent, Edinburgh, EH16 4TJ, UK

**Keywords:** glucocorticoids, 5α-reductases, adrenal insufficiency, HPA axis

## Abstract

Patients with critical illness or hepatic failure exhibit impaired cortisol responses to ACTH, a phenomenon known as ‘relative adrenal insufficiency’. A putative mechanism is that elevated bile acids inhibit inactivation of cortisol in liver by 5α-reductases type 1 and type 2 and 5β-reductase, resulting in compensatory downregulation of the hypothalamic–pituitary–adrenal axis and adrenocortical atrophy. To test the hypothesis that impaired glucocorticoid clearance can cause relative adrenal insufficiency, we investigated the consequences of 5α-reductase type 1 deficiency in mice. In adrenalectomised male mice with targeted disruption of 5α-reductase type 1, clearance of corticosterone was lower after acute or chronic (eightfold, *P*<0.05) administration, compared with WT control mice. In intact 5α-reductase-deficient male mice, although resting plasma corticosterone levels were maintained, corticosterone responses were impaired after ACTH administration (26% lower, *P*<0.05), handling stress (2.5-fold lower, *P*<0.05) and restraint stress (43% lower, *P*<0.05) compared with WT mice. mRNA levels of *Nr3c1* (glucocorticoid receptor), *Crh* and *Avp* in pituitary or hypothalamus were altered, consistent with enhanced negative feedback. These findings confirm that impaired peripheral clearance of glucocorticoids can cause ‘relative adrenal insufficiency’ in mice, an observation with important implications for patients with critical illness or hepatic failure, and for patients receiving 5α-reductase inhibitors for prostatic disease.

## Introduction

Activation of the hypothalamic–pituitary–adrenal (HPA) axis is a vital component of the stress response, driving production of glucocorticoid hormones (cortisol in humans, corticosterone in rodents) that mediate essential adaptations of the immune, metabolic and cardiovascular systems in response, for example, to infection or injury. Paradoxically, low cortisol concentrations have been observed in patients with critical illness, particularly after administration of synthetic adrenocorticotrophic hormone (ACTH); this phenomenon has been termed ‘relative adrenal insufficiency’ and has led patients being treated with hydrocortisone (Annane *et al*. 2002). Similarly impaired cortisol responses to synthetic ACTH have been observed in patients with liver disease (Harry *et al*. 2002, Fernandez *et al*. 2006, Tsai *et al*. 2006). The mechanisms involved in these altered cortisol responses are complex, with abnormalities observed in central HPA axis drive, adrenal steroidogenesis and plasma protein binding (Cooper & Stewart 2009). In addition, recent observations have indicated that a key contribution results from alterations in the enzymatic clearance of cortisol. In liver disease, inactivation of cortisol by the enzymes 5β-reductase (*Akr1d1*) and 11β-hydroxysteroid dehydrogenase type 2 (*Hsd11b2*; Frey 2006, McNeilly *et al*. 2010) is impaired. In critical illness, we have recently reported profoundly impaired clearance of cortisol, which was attributed to impaired 5α-reductase and 5β-reductase activity in liver and *Hsd11b2* in kidney (Boonen *et al*. 2013). We also demonstrated an association between impaired cortisol clearance and a reduced cortisol response to synthetic ACTH. As endogenous ACTH levels were paradoxically low in critically ill patients, we proposed the hypothesis that substantially slower clearance of cortisol results in enhanced negative feedback suppression of ACTH and hence compensatory downregulation of cortisol production to maintain cortisol levels (Boonen *et al*. 2013). As a result, adrenal atrophy ensues and the incremental response to exogenous ACTH is impaired. However, in disease settings, it is difficult to confirm the primary cause of changes in HPA axis function.

An influence of peripheral steroid metabolism on HPA responses has been demonstrated in mice unable to regenerate or inactivate active glucocorticoid by *Hsd11b1* or *Hsd11b2* respectively (Kotelevtsev *et al*. 1997, 1999, Harris *et al*. 2001, Carter *et al*. 2009). Patients with impaired cortisol clearance due to *Hsd11b2* deficiency have reduced total cortisol production, presumably through suppression of the HPA axis (Stewart *et al*. 1988), which is mirrored in *Hsd11b2* knockout mice (Kotelevtsev *et al*. 1999). In contrast, mice unable to regenerate tissue glucocorticoids due to deletion of *Hsd11b1* or hexose-6-phosphate dehydrogenase exhibit adrenal hypertrophy (Kotelevtsev *et al*. 1997, Harris *et al*. 2001, Lavery *et al*. 2006, Carter *et al*. 2009). This again is consistent with the clinical scenario, where enhanced cortisol clearance in patients with rare *Hsd11b1* or hexose-6-phosphate dehydrogenase deficiency results in increased ACTH drive to the adrenals, and hence elevated adrenal androgens (Taylor *et al*. 1984, Phillipov *et al*. 1996, Jamieson *et al*. 1999, Draper *et al*. 2003).

5α-Reductases are responsible for one-third to a half of total peripheral glucocorticoid clearance, but their participation in the regulatory feedback loops mediated via the HPA axis has not been studied. Upregulation of 5α-reductase has been invoked to explain adrenal androgen excess in polycystic ovary syndrome (Stewart *et al*. 1990, Fassnacht *et al*. 2003, Tsilchorozidou *et al*. 2003). Although congenital deficiency of 5α-reductase is rare (Imperato-McGinley *et al*. 1974), an increasing number of men are prescribed 5α-reductase inhibitors for benign prostatic hyperplasia. The consequences, if any, for the HPA axis are unknown. We tested, in a mouse model, whether deficiency of 5α-reductase 1 (*Srd5a1*) activity causes relative adrenal insufficiency.

## Materials and methods

Chemicals were obtained from Sigma unless otherwise stated.

### *In vivo* protocols

Mouse embryos with targeted disruption of *Srd5a1* (Mahendroo *et al*. 1996, 1997) (C57Bl6/SvEv/129 mixed background; Jackson Laboratory, Bar Harbor, ME, USA), were re-derived (University of Edinburgh) and heterozygote offspring crossed to generate homozygote male ‘WT’ and ‘knock-out’ (*Srd5a1*-KO) mice that were used for study at 4–5 months of age. All experiments were carried out under UK Home Office license and mice were housed singly for 1 week prior to investigations. Mice were maintained with *ad libitum* access to standard chow (Special Diet Services, Witham, UK) under regulated conditions of light and darkness (lights on from 0700 to 1900 h). Following killing by decapitation, trunk blood was collected and one adrenal gland and the thymus gland were removed and formalin-fixed. The hypothalamus, remaining brain, pituitary and other adrenal were frozen on soft dry ice. All samples were stored at −80 °C.

#### Glucocorticoid clearance

Weight matched (approximately 30 g) male WT and *Srd5a1*-KO mice were adrenalectomised under isoflurane anaesthesia to remove endogenous glucocorticoids (*n*=8/group). After 2 weeks recovery, mice received corticosterone (2 μg, in 10% ethanol with 0.025% β-cyclodextrin) by s.c. bolus injection and blood samples were obtained by tail-tip bleed after 0, 15, 30, 60 and 90 min.

In another group of mice, osmotic minipumps were implanted subcutaneously during adrenalectomy to administer corticosterone for 3 weeks before mice were killed (100 μg/day corticosterone in 1:1 DMSO:propylene glycol; Alzet model 2004, Charles River UK Ltd, Margate, Kent, UK). Clearance of corticosterone was calculated as the infusion rate divided by the steady-state plasma concentrations determined in trunk blood samples collected at killing.

Further intact WT and *Srd5a1*-KO mice (*n*=12/group) received corticosterone (50 μg/day) for 2 weeks by minipump as indicated earlier. Mice were killed by decapitation and trunk blood was collected.

#### Responses to stress

Mice (*n*=8/group) were killed 10 min following cage disturbance and handling. Trunk blood was collected and brains were harvested.

The remaining mice (*n*=12–18/genotype) were removed from their home cage at 0800 h with minimum disruption and blood was obtained rapidly (within 30 s) by tail-nick. The animals were subsequently restrained within Plexiglas restraint tubes for 15 min and another set of blood samples was taken. They were returned to their home cages and a further blood samples obtained either at 30, 60 or 90 min following the start of the restraint stress (*n*=6/time point). Blood samples were collected at only one time point following the end of the restraint stress to avoid confounding effects of repeated stress.

#### Responses to dexamethasone and ACTH

Male mice (*n*=9–10/genotype) received an injection of dexamethasone (not a substrate for *Srd5a1* (McGuire *et al*. 1960), 10 μg/kg body weight intra-peritoneally) at 1300 h to achieve partial (approximately 50%) suppression of the HPA axis. Blood samples were obtained by tail-nick at 1500 h, after which mice received injections of either vehicle (saline) or ACTH (Synacthen diluted in saline, Alliance, Chippenham, UK, 0.1 μg/kg; EC_50_) and blood samples were again collected after 30 min.

### Laboratory protocols

#### Morphometric analysis of adrenal glands

Fixed adrenal glands (*n*=8–14) were sectioned (5 μm), stained with haematoxylin and eosin and examined by light microscopy (Zeiss Axioscope, 40× magnification). The cells were photographed using a Cool Snap Photometrics camera and the number of cells in a given area (∼160 μm^2^ for the inner and outer zona fasciculata and 40 μm^2^ for the zona glomerulosa and zona reticularis) from each adrenal was counted using MCID 7.0 software. The adrenals were analysed in a randomised order by an observer blinded to the genotype. Two areas were examined in two slices from each adrenal and the mean values were calculated. Cryosections (10 μm) of frozen adrenals were stained with Oil Red O to visualise lipid distribution and examined by microscopy.

#### Quantification of mRNAs by real-time quantitative PCR

Adrenals (*n*=11–14/genotype), hypothalami and pituitaries (*n*=6/genotype) were homogenised using a QIAshredder column (Qiagen Ltd) and total RNA was extracted from snap-frozen tissue samples using the Qiagen RNeasy system, and 500 ng total RNA were reverse transcribed into cDNA with random primers using the QuantiTect DNase/reverse Transcription Kit. cDNA (equivalent to 1 ng total RNA) was incubated in triplicate with gene-specific primers and fluorescent probes (Table 1: Applied Biosystems or the Universal Probe Library, Roche Diagnostics) in 1× Roche LightCycler 480 Probes mastermix. PCR cycling and detection of fluorescent signal were carried out using a Roche LightCycler480. A standard curve was constructed for each set of primer probes using a serial dilution of cDNA pooled from all samples. The results were corrected for the mean of abundance of reference genes (*Gapdh* and *Tbp* in the hypothalamus, *Rn18s*, *Tbp* and *Actb* mRNAs for adrenal with *Ppia* in addition for pituitary); the respective means of the reference genes did not differ between groups.

#### Quantification of mRNAs by *in situ* hybridisation

Cryosections (10 μm) of brains (*n*=8–9/group) were mounted onto electrostatic slides. Antisense and sense riboprobes for *Nr3c1*, *Nr3c2* and *Crh* transcripts were prepared as described previously (Harris *et al*. 2001). Tissues were processed and hybridised, probes visualised by autoradiography and quantified using a microcomputer imaging system operated by Zeiss KS300 3.0 computer software. *Nr3c1* and *Nr3c2* transcripts were quantified in the dentate gyrus (DG), CA1, CA2, CA3 and CA4 regions of the hippocampus and *Crh* transcripts in the paraventricular nucleus of the hypothalamus PVN, by counting of the number of silver grains in each region by a blinded observer, reporting the mean counts from six randomly selected areas (radius=43 μm) minus background count.

#### Biochemical assays

Velocities of hepatic *Akr1d1* and *Hsd11b1* were quantified in tissue homogenates as reported previously (Livingstone *et al*. 2009). Plasma corticosterone was measured by an in-house RIA, without significant cross-reactivity with dexamethasone (Holmes *et al*. 1995), and ACTH by RIA (MP Biomedical, California, CA, USA).

To quantify tissue corticosterone, liver (approximately 300 mg) was homogenised (3 ml, 7:2 methanol:water) and enriched with 125 ng epi-cortisol as internal standard. The homogenates were shaken (15 min) and centrifuged (3200 ***g***, 45 min at 4 °C), the supernatant was reduced to dryness under oxygen-free nitrogen (OFN, 60 °C) and then dissolved in 1:1 methanol:hexane (20 ml). The methanolic layer was reduced to dryness under OFN; the residue was dissolved in water (400 μl) and steroids were extracted with ethyl acetate (ten volumes). Organic extracts were dried under OFN; the residue was dissolved in 30% methanol (5 ml) and then applied to Megabond columns (2 g, C18, Varian, Oxford, UK) and steroids were eluted with methanol (5 ml). The eluate was dissolved in mobile phase (methanol:water 50:50 containing 0.1% formic acid) and steroids were analysed by liquid chromatography tandem mass spectrometry using a Surveyor pump and TSQ Quantum Discovery triple quadropole mass spectrometer (Thermo Electron, Hemel Hempstead, UK). Steroids were separated using a Kinetex PFP column (100×3 mm, 2.6 μm, 15 °C; Phenomenex, Macclesfield, UK) using gradient elution; initial conditions of methanol:water 50:50, each with 0.1% formic acid, were maintained for 1 min and then programmed to achieve 35:65 (1–2.5 min), 30:70 (2.5–6 min) and 10:90 (6–6.5 min). The mass spectrometer was operated in a positive electrospray ionisation mode with selected reactions monitoring (transition, collision energy, tube lens: corticosterone; *m/z* 347→121, 27 V, 103 V epi-cortisol *m/z* 363→121, 30 V, 95 V). The peak areas were integrated using Xcalibur software (Thermo Electron) and corticosterone was quantified as a proportion of epi-cortisol as internal standard against a calibration curve. The limit of detection was less than 1 ng corticosterone. Corticosterone concentrations are presented corrected for total tissue weight. For brain steroid measurement, half brains (sagitally sectioned) were homogenised in 1 ml ethyl acetate:ethanol (1:1 v/v), the homogenate was dripped into 10 ml ice-cold ethanol:acetic acid:water (95:3:2 v/v) and incubated at −20 °C overnight. The samples were then processed in the same way as for liver homogenates.

### Statistical analysis

Data are mean±s.e.m. and were compared by Student's *t*-test or repeated measure ANOVA with Fisher's *post hoc* test as appropriate. Area under the curve was calculated using Kinetica software (Thermo Electron). *P*<0.05 was considered statistically significant.

## Results

### *Srd5a1*-KO mice have decreased clearance of corticosterone

**Figure 1:**
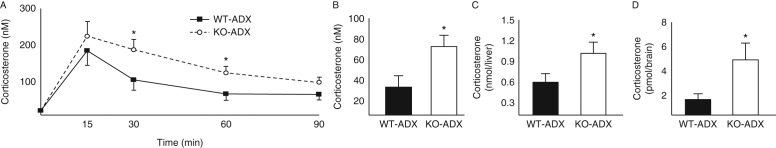
Clearance of corticosterone in *Srd5a1*-KO and WT mice. Plasma corticosterone concentrations following (A) corticosterone bolus injection and (B) chronic infusion were higher in adrenalectomised mice deficient in 5α-reductase 1 (KO, open circles/white bars) than in WT mice (WT, black squares/bars). Amounts of corticosterone in (C) liver and (D) brain were higher in KO than in WT mice following chronic infusion of corticosterone. *P<0.05 versus WT.

Clearance of corticosterone was slower in *Srd5a1*-KO mice compared with WT mice (3.42±0.61 vs 30.15±11.75 ml/min; *P*<0.05), demonstrated by higher circulating corticosterone concentrations after both an acute bolus (Fig. 1A) and chronic infusion (Fig. 1B) of corticosterone in adrenalectomised mice. Residual corticosterone was detectable in adrenalectomised mice, but the levels were not different between genotypes. Slower clearance was also reflected in higher liver (Fig. 1C) and brain (Fig. 1D) corticosterone concentrations in *Srd5a1*-KO mice following chronic corticosterone infusion. The velocities of hepatic *Hsd11b1* and *Akr1d1* were not different between *Srd5a1*-KO and WT mice (11.7±0.8 vs 13.4±0.5 nmol/mg per h, 244±22 vs 299±32 pmol/mg per h, respectively), and this was also reflected in the abundances of the transcript for *Hsd11b1* (0.86±0.16 vs 1.05±0.11), although mRNA encoding *Akr1d1* was present at lower levels in *Srd5a1*-KO mice than in WT controls (0.54±0.03 vs 0.90±0.15; *P*=0.04).

### Resting plasma corticosterone levels are maintained in intact *Srd5a1*-KO mice

**Figure 2:**
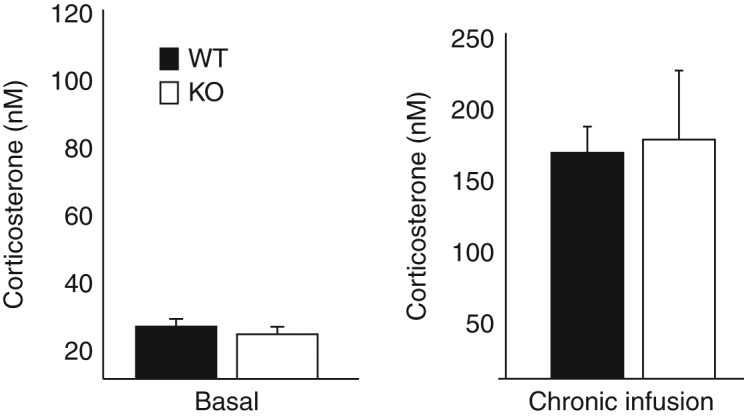
Plasma corticosterone in *Srd5a1*-KO and WT mice. Basal plasma corticosterone concentrations were the same in (A) unstressed mice of both genotypes and (B) after chronic infusion of corticosterone. *n*=6–8/group per treatment compared by Student's *t*-tests. WT (white bars); KO, *Srd5a1*-deficient mice (black bars).

There were no differences between *Srd5a1*-KO and WT mice in basal (unstressed) circulating corticosterone at the diurnal nadir (Fig. 2A). Intact *Srd5a1*-KO mice adapted appropriately to corticosterone infused at sub-physiological replacement dose, maintaining the same total circulating corticosterone concentrations as WT mice (Fig. 2B). The ACTH level measured in trunk blood was not different between *Srd5a1*-KO and WT mice (208.4±49.4 vs 125.5±24.3 pg/ml; *P*=0.13, *n*=17–18).

**Figure 3:**
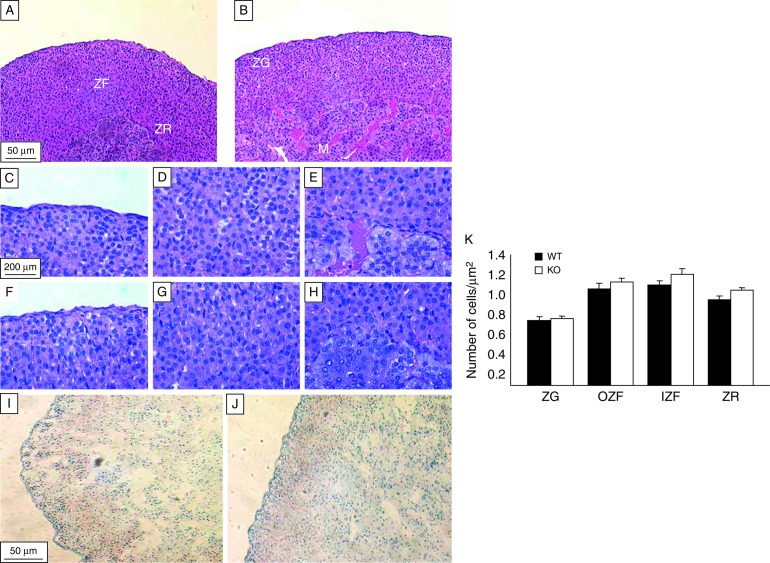
Morphological analysis of adrenal glands from WT and 5αR1-KO mice. Representative sections (5 μm) of adrenal glands stained with haematoxylin and eosin. Gross histological (10× magnification) differences between (A) WT mice and (B) those deficient in 5α-reductase 1 (KO) were not observed. Individual zones are shown at higher magnification (40×): (C and F) zona glomerulosa; (D and G) zona fasciculata and (E and H) zona reticularis in WT and KO mice respectively. Panels I (WT) and J (KO) show representative sections (5 μm) of frozen adrenal glands stained with Oil red O and photographed at 10× magnification; differences in lipid accumulation were not observed. Panel K shows the number of cells per zone of the adrenal gland in WT (black bars) and KO (white bars) mice. Significant changes in the number of cells in any of the adrenal zones were not observed between genotypes. M, adrenal medulla; ZG, zona glomerulosa; ZF, zona fasciculata; IZF, inner zona fasciculata; OZF, outer zona fasciculata; ZR, zona reticularis. *n*=6–8/group per treatment.

Weights of the adrenal and thymus glands were not different between genotypes (Table 2). Gross morphometric examination of the adrenal glands did not reveal any differences (Fig. 3A and B). Neither the number of cells per unit area (Fig. 3C, D, E, F, G, H, and K), the distribution of lipid by Oil Red O staining (Fig. 3I and J), nor the abundance of adrenal mRNA encoding *Cyp11b1* (0.46±0.05 vs 0.43±0.05) differed between genotypes.

### 5αR1-KO mice exhibit ‘relative adrenal insufficiency’ during stimulation by stress or ACTH

**Figure 4:**
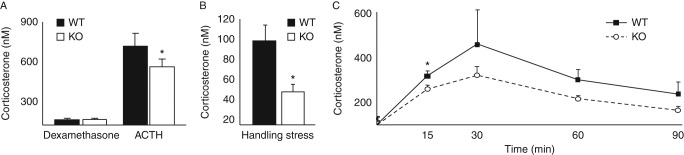
Dynamic responses of the hypothalamic–pituitary–adrenal axis to stimulation and suppression. (A) Circulating concentrations of corticosterone were not different between mice deficient in *Srd5a1* (KO, open circles/white bars) and WT mice (WT, black squares/bars), following partial suppression of the hypothalamic–pituitary–adrenal axis with dexamethasone, but the corticosterone response to ACTH stimulation was attenuated in KO mice compared with WT (by two-way ANOVA, *P*<0.01 for effect of ACTH and *P*<0.05 for effect of genotype). (B) Circulating corticosterone levels were lower in KO mice following mild handling stress than in WT mice. (C) The increase in corticosterone in response to 15 min acute restraint stress (indicated by dark bar on graph) was attenuated in KO mice (open circles) compared with WT mice (black squares) (*n*=12–18/genotype at *t*=0 and 15 and *n*=6 at other time points; *P*<0.01 for change with time and *P*<0.05 for genotype effect by repeated measures ANOVA). Unless stated, *n*=6–8/group per treatment. *P<0.05 vs WT.

Corticosterone levels were suppressed to a similar degree in response to a sub-maximal dose of dexamethasone (Fig. 4A). Following exogenous ACTH administration, the increase in plasma corticosterone concentrations was attenuated in *Srd5a1*-KO mice compared with WT (Fig. 4A). Circulating corticosterone levels were lower in *Srd5a1*-KO than in WT mice, in response to both minor stress (cage disturbance and handling; Fig. 4B) and after restraint stress (Fig. 4C; area under the curve for plasma corticosterone (10.3±2.2 vs 18.0±2.0 μM.min, *P*=0.003).

### The molecular control of the HPA axis compensates for altered corticosterone clearance in *Srd5a1*-KO mice

**Figure 5:**
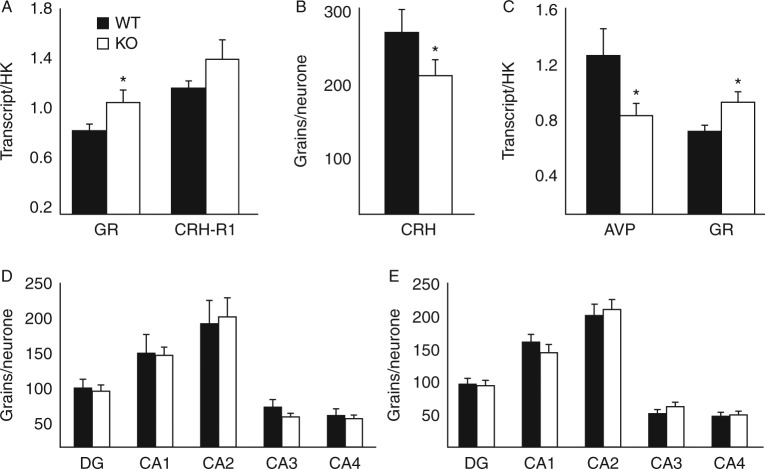
Transcript abundances of genes regulating the hypothalamic–pituitary–adrenal axis. (A) In pituitary, abundance of transcripts for glucocorticoid receptor (GR) but not corticotrophin releasing hormone receptor 1 (*Crhr1*) was higher in mice deficient in *Srd5a1* (KO; black bars) compared with WT controls (WT; white bars). In the paraventricular nucleus of the hypothalamus, the transcripts of (B) *Crh* and (C) arginine–vasopressin (*Avp*) were lower in KO mice compared with WT, while *Nr3c1* transcript was higher. In the CA1, CA2, CA3, CA4 regions or dentate gyrus (DG) of the hippocampus, differences were not observed between genotypes in abundances of transcripts of (D) *Nr3c1* or (E) mineralocorticoid receptors (*Nr3c2*). Data are mean±s.e.m., HK, housekeeping genes; *n*=6–9/group. *P<0.05 vs WT.

Pituitary and brain transcript abundances are shown in Fig. 5. In *Srd5a1*-KO mice, glucocorticoid receptor (*Nr3c1*) transcripts were more abundant in the pituitary and hypothalamus but not in any regions of the hippocampus (Fig. 5A, C, and D). Mineralocorticoid receptor (*Nr3c2*) transcripts in the hippocampus were also unaffected by genotype (Fig. 5E). Corticotrophin-releasing hormone receptor 1 (*Crh-r1*) transcript abundance in pituitary was not different between genotypes (Fig. 5A); however, *Crh* mRNA in the PVN (Fig. 5B) and arginine–vasopressin (*Avp*; Fig. 5C) mRNAs in the hypothalamus were both lower in abundance in 5αR1-KO than in WT mice. Representative images of the *in situ* analysis are shown in Supplementary Figure 1, see section on supplementary data given at the end of this article.

## Discussion

These data confirm that the enzyme *Srd5a1* contributes in large part to glucocorticoid clearance in mice, and that its targeted disruption is sufficient to induce a phenotype analogous to ‘relative adrenal insufficiency’ in patients with impaired cortisol clearance. Although baseline plasma ACTH and corticosterone are normal, mice lacking *Srd5a1* exhibit not only impaired corticosterone responses to ACTH but also impaired responses to mildly and moderately stressful stimuli. Moreover, this occurred in the absence of any demonstrable primary histological abnormality in the adrenal glands and was associated with alterations in neuroendocrine signalling pathways in the hypothalamus and pituitary that are most readily explained by enhanced negative feedback by corticosterone resulting from its impaired peripheral clearance and causing a compensatory downregulation of the HPA axis.

*Srd5a1* is present in high abundance in murine liver, similar to the situation in humans, and therefore deficiency at this site predicts impaired peripheral metabolism, as observed in the experiment involving chronic infustion of corticosterone. However 5α-reductases are expressed also in brain (Lephart 1993a), specifically in the hypothalamus (Thigpen *et al*. 1993, Lund *et al*. 2006) (where *Srd5a1* is more abundant than *Srd5a2* (Lephart 1993b)) and to a lesser extent in pituitary (Lephart 1993b). Deficiency or inhibition of 5αR1 at these sites may attenuate local inactivation of glucocorticoids, and thereby enhance negative feedback suppression of the HPA axis independently of the altered peripheral clearance. Following chronic infusion of corticosterone, we did not find a disproportionate elevation in brain corticosterone levels in *Srd5a1*-KO mice; the elevation in brain corticosterone levels was of a similar magnitude to the higher plasma corticosterone levels in *Srd5a1*-KO mice. Furthermore, the observation that plasma ACTH in unstressed animals is not suppressed by 5αR1 deficiency indicates that the primary driver for altered HPA axis function lies in altered peripheral corticosterone clearance rather than failure of corticosterone inactivation in the brain.

The modulatory role of ligand concentrations on glucocorticoid and mineralocorticoid receptors (*Nr3c1* and *Nr3c2*) at feedback sites within the HPA axis has been studied in other rodent models. In the extreme scenario of adrenalectomy, mice have dramatically higher ACTH levels, along with greater *Nr3c1* abundance at feedback sites (Han *et al*. 2007), the receptor apparently auto-regulating in response to absence of ligand (Herman & Spencer 1998, Ing 2005). The converse is true with chronic stress associated with elevated glucocorticoids, suppressing GR levels in the PVN but not in the hippocampus (Makino *et al*. 1995). However, in models of more subtle modulation of ligand, e.g. in mice with increased peripheral clearance induced by *Hsd11b1* deficiency, changes in *Nr3c1* expression may not auto-regulate in the same manner. On a 129/MF1 genetic background, mice lacking *Hsd11b1* develop a greater degree of adrenal hypertrophy (approximately 70%) (Harris *et al*. 2001) than on a C57BL/6 background (approximately 20%) (Carter *et al*. 2009). This appears to be attributable to C57BL/6 mice being better able than 129/MF1 mice to upregulate *Nr3c1* at central feedback sites, contributing to resetting of the HPA axis (Carter *et al*. 2009). Specifically, in the mixed strain 129/MF1 *Hsd11b1*-deficient mice with more pronounced HPA abnormalities, exaggerated peripheral glucocorticoid clearance was accompanied by suppressed *Nr3c1* expression in the PVN. Against this background, our findings are consistent with impaired peripheral glucocorticoid metabolism in *Srd5a1*-KO mice bred on a mixed genetic background (comprising C57Bl6/SvEv/129), with an upregulation of *Nr3c1* transcript in central feedback sites (Mahendroo *et al*. 1997). Increased *Nr3c1* action may be associated with downregulation of transcription of *Crh* (Malkoski & Dorin 1999) and *Avp* (Burke *et al*. 1997) mRNAs in the hypothalamus, together mediating a compensatory downregulation of stress-induced ACTH secretion. Similar downregulation of hypothalamic *Crh* transcripts has been demonstrated *in vivo* in mice with increased gene dosage of *Nr3c1* (Reichardt *et al*. 2000). However, any change in *Nr3c1*-mediated signalling with manipulation of *Srd5a1* appears too subtle to be detected by dexamethasone suppression testing in mice. Furthermore, basal circulating corticosterone levels were unaltered by disruption of *Srd5a1*, and intact mice adapted appropriately to the infusion of low physiological levels of glucocorticoid. Overall these data indicate that a centrally driven primary abnormality of the HPA axis causing relative adrenal insufficiency is unlikely and that the phenotype is most probably driven by impaired peripheral metabolic clearance of corticosterone.

Although our investigations focused on glucocorticoids, 5α-reductases also metabolise a wide range of other steroid hormones, some of which may have important effects on the CNS. Androgens can repress CRH (Bao *et al*. 2006), but lack of the most potent androgen 5α-dihydrotestosterone (DHT) would predict a more, rather than less, dynamic HPA axis with deficiency of *Srd5a1*. Reduction of testosterone to DHT by *Srd5a1* is also thought to regulate the feedback responses of GNRH neurons to androgens (Tobyn & Canny 1998), which we did not investigate here. 5α-Reduced neurosteroids (e.g. allopregnanolone) have been implicated in the attenuation of behavioural responses to anxiety, acting via the GABA-A receptors to reduce neuronal excitability; this may impinge upon the HPA axis and attenuate stress responses, as is most apparent during late pregnancy (Russell & Brunton 2006).

These findings may be relevant to clinical practice. It is notable that in *Srd5a1*-KO mice corticosterone clearance was reduced by approximately 80% compared with that of WT mice; this was attributed to changes in 5α-reduction as the velocities of hepatic *Akr1d1* and *Hsd11b1* were unaltered (despite a modest reduction in *Akr1d1* mRNA). Findings in *Srd5a1*-KO mice were therefore rather larger in magnitude than the approximately 50% reduction in cortisol clearance we demonstrated in critically ill patients during infusion of deuterated-cortisol (Boonen *et al*. 2013), especially considering that critically ill patients have ‘extra’ loss of cortisol-clearing capacity attributed to reduced activity of *Akr1d1* and *Hsd11b2*. It may be that *Srd5a1* contributes more to glucocorticoid clearance in mice than it does in humans, which is plausible as 5α-reductase type 1 is the only isozyme expressed in mouse liver (Mahendroo *et al*. 1996, 1997) whereas human liver contains both 5α-reductase type 1 and type 2 (Evans & Goa 2003).

These data indicate that there may be a risk of inadequate cortisol responses to stress in men taking 5α-reductase inhibitors for prostatic disease, particularly when the dual 5α-reductase type 1 plus type 2 inhibitor, dutasteride, is prescribed in preference to the selective type 2 inhibitor, finasteride. Previous investigations following inhibition of 5α-reductases in humans indicate that women, who are sometimes treated with finasteride for hirsutism, do exhibit changes in the HPA axis (Fruzzetti *et al*. 1994), at least for a short-term period, with decreased basal plasma cortisol and an impaired cortisol response to exogenous ACTH. However, studies in men have shown no change in basal cortisol (Rittmaster *et al*. 1994, Lewis *et al*. 1997, Uygur *et al*. 1998) or in response to high dose ACTH (Rittmaster *et al*. 1994), although a lower, potentially more discriminatory dose has not been tested. Administration of 5α-reductase inhibitors to men suppresseses daily total glucocorticoid production rates by approximately 20% (Upreti *et al.* 2014), but the proportion of cortisol excreted in urine as 5α-reduced metabolites is substantially higher in women than in men (Andrew *et al*. 1998), consistent with 5α-reductases making a greater contribution to clearance of cortisol. In women, this may be attributable to *Srd5a2* rather than *Srd5a1* in liver, because effects on the HPA axis consistent with altered cortisol clearance were observed with the selective type 2 inhibitor finasteride (Fruzzetti *et al*. 1994). Thus, women may be more susceptible than men to ‘relative adrenal insufficiency’ if given a 5α-reductase inhibitor.

In conclusion, our findings emphasise that peripheral steroid metabolism has potentially potent effects on the HPA axis. In mice with life-long deficiency of *Srd5a1*, adrenal responsiveness to stress is substantially impaired. This may have implications for individuals receiving 5α-reductase inhibitors chronically, and particularly for women in whom the 5α-reductase pathway of cortisol metabolism is thought to be of greater significance than in men. Our data confirm the hypothesis that ‘relative adrenal insufficiency’ can result from impaired glucocorticoid clearance, which appears to be relevant in many clinical scenarios, including hepatic failure and critical illness.

## Supplementary data

This is linked to the online version of the paper at http://dx.doi.org/10.1530/JOE-13-0563.

## Author contribution statement

The authors have made the following declarations about their contributions: D E W L designed and performed experiments, analysed and interpreted data and prepared the manuscript; E M D, C Y, L E C, J A M, M K and K A H, performed experiments and analysed and interpreted data; C J K, B R W, R A analysed and interpreted data and prepared the manuscript.

## Figures and Tables

**Table 1:** Assay details for real-time PCR. ABI assay numbers: cyclophilin (*Ppia*), Mm02342429_g1; corticotrophin-releasing hormone receptor 1 (*Crhr1*), Mm00432670_m1; 11β-hydroxylase (*Cyp11b1*), Mm01204952_m1

	**Forward primer**	**Reverse primer**	**UPL probe no.**
18S ribosomal RNA (*Rn18s*)	ctcaacacgggaaacctcac	cgctccaccaactaagaacg	77
β-actin (*Actb*)	ctaaggccaaccgtgaaaag	accagaggcatacagggaca	64
5α-reductase 1 (*Srd5a1*)	gggaaactggatacaaaataccc	ccacgagctccccaaaata	41
5α-reductase 2 (*Srd5a2*)	cgcacattacttccacagga	cagaaagatcaccgctgataaa	34
5β-reductase (*Akr1d1*)	gaaaagatagcagaagggaaggt	gggacatgctctgtattccataa	79
11β-hydroxysteroid dehydrogenase 1 (*Hsd11b1*)	tctacaaatgaagagttcagaccag	gccccagtgacaatcacttt	1
Glucocorticoid receptor (*Nr3c1*)	gacgtgtggaagctgtaaagt	catttcttccagcacaaaggt	56
Arginine vasopressin (*Avp*)	gctgccaggaggagaactac	aaaaccgtcgtggcactc	84
TATA box-binding protein (*Tbp*)	gggagaatcatggaccagaa	gatgggaattccaggagtca	97
GAPDH	gggttcctataaatacggactgc	ccattttgtctacgggacga	52

**Table 2:** Characteristics of *Srd5a1*-KO and WT mice. Data are mean±s.e.m. *n*=12–16/group

	**WT**	**5αR1-KO**
Body weight (g)	26.3±0.85	27.0±0.76
Adrenal weight (mg)	2.2±0.08	2.1±0.09
Adrenal weight/body weight (mg/g)	0.17±0.01	0.16±0.01
Thymus weight (mg)	40.4±3.3	39.2±3.5

*Srd5a1*-KO, 5α-reductase 1 knockout.
